# Electrodeposition of Pt-Ni nanoparticles on graphene as an electrocatalyst for oxygen reduction reaction

**DOI:** 10.3389/fchem.2022.1061838

**Published:** 2022-10-24

**Authors:** Siming Li, Xuerong Yan, Meng Shi, Pengfei Wei, Haigang Lu, Zhiyang Zhang, Yong Zhang, Yawei Li

**Affiliations:** ^1^ School of Chemistry and Chemical Engineering, Shanxi University, Taiyuan, China; ^2^ Institute of Molecular Science, Shanxi University, Taiyuan, China; ^3^ Key Lab of Materials for Energy Conversion and Storage of Shanxi Province, Shanxi University, Taiyuan, China; ^4^ CAS Key Laboratory of Coastal Environmental Processes and Ecological Remediation, Research Center for Coastal Environmental Engineering and Technology, Yantai Institute of Coastal Zone Research, Chinese Academy of Sciences, Yantai, China

**Keywords:** electrocatalyst, electrodeposition, carbon support, oxygen reduction, graphene

## Abstract

Owing to its novel properties, such as high electrical conductivity and large specific surface area, graphene has been found as suitable support material for the electrocatalyst design. This work reports the preparation of platinum-nickel alloy nanoparticles (PtNi NPs) electrocatalyst by electrodeposition of PtNi NPs onto graphene support. The obtained PtNi/graphene electrocatalysts were characterized by high resolution transmission electron microscopy (HRTEM), energy-dispersive X-ray microscopy (EDX), X-ray diffraction (XRD), X-ray photoelectron spectroscopy (XPS), and thermogravimetric analysis (TGA) indicating the controllable morphological and compositional profiles of PtNi NPs on graphene. The electrocatalytic characteristics of PtNi/graphene toward oxygen reduction reaction (ORR) were systematically investigated showing comparable kinetic performance. Moreover, the graphene during electrodeposition process induces carbon vacancies and defects, increasing interaction between nanoparticles and graphene and enhancing electrocatalytic stability by limiting aggregation of the nanoparticles during accelerated stability test. This work opens a promising path for the preparation of graphene-supported alloy electrocatalyst.

## 1 Introduction

Electrochemical energy conversion and storage devices are critical enabling technologies for carbon-neutral renewable energy (Gasteiger and Markovic, 2009). These systems are required to be optimized in terms of cost, efficiency and longevity to integrate into consumer and industrial applications (Gittleman et al., 2019). For many of these devices, including fuel generators (electrolyzers) and fuel consumers (fuel cells), the limiting factor for their efficiency and operational lifetime directly depends on performance of the electrocatalysts on the electrodes (Seh et al., 2017; Li et al., 2020). Although notable achievement has been made on non-platinum group metal (PGM) catalysts (Xie et al., 2020), supported Pt-based catalyst is still the most efficient and commonly used cathodic catalyst for oxygen reduction reaction (ORR) in polymer electrolyte membrane (PEM) fuel cell (Tian et al., 2020). To date, while major research efforts have been underway to develop Pt-based metals to increase the active sites number and intrinsic activities through their morphological and compositional optimizations (Snyder et al., 2012; Seh et al., 2017; Li et al., 2019), it must been recognized that support materials, by maintaining good catalyst-support interaction and reactants/products transport (Jha et al., 2008), are also vital and highly influential in determining the performance, longevity and cost effectiveness of the electrocatalyst (Sharma and Pollet, 2012). The choice of support material to build good interaction with the catalyst is not only to improve catalyst efficiency and life time but also govern charge transfer (Sharma and Pollet, 2012). Therefore, a wide category of nanostructured carbon based materials has been investigated as catalyst supports for ORR, such as carbon blacks (Dicks, 2006), mesoporous carbon (Yarlagadda et al., 2018), carbon nanotubes (Knupp et al., 2008), carbon nanofiber (Sebastián et al., 2012), and graphene (Kou et al., 2011). The design principle of these carbon nanomaterials applicable in electrocatalyst support is high specific surface area for the dispersion of metal catalyst, high electrical conductivity for electrochemical reactions, optimized carbonaceous structures for transferring reactants/products, and good thermal/chemical stability for catalytic durability (Sharma and Pollet, 2012).

As an atomically thin sheet of hexagonally arranged carbon atoms which offer fast electron transferring, graphene has attracted a lot of interest for various applications (Higgins et al., 2016). The unique structure of two-dimensional planner structure composed of sp (Gittleman et al., 2019)-bonded carbon atoms with one-atomic thickness enables superior electric conductivities to the carbon and allows both the edge planes and basal planes to interact with the metal nanoparticles (Higgins et al., 2016). Owing to these outstanding electrical and mechanical properties, graphene has been found as suitable support material for the electrocatalyst design (Yoo et al., 2009; Soin et al., 2011; Niu et al., 2012; Suh et al., 2016). Recent progress in preparation techniques has made it possible to incorporate metal catalyst with graphene and study the properties experimentally (Higgins et al., 2016). Many literatures suggested that the electrochemical performance of graphene-supported electrocatalyst is highly sensitive to the carbon supporting method (Sharma and Pollet, 2012). Soin et al. used vertically aligned graphene nanoflakes (FLGs) as Pt nanoparticle support for electrocatalysis application. The FLGs were synthesized using microwave plasma enhanced chemical vapor deposition method and the Pt nanoparticles were deposited using sputtering technique. Fast electron transfer kinetics were demonstrated resulting from the highly graphitized edge structure of FLG nanoflakes (Soin et al., 2011). Kou et al. (2011) reported a new method to deposit catalyst by forming metal-metal oxide-graphene triple-junction structure where the defects and functional groups on graphene play an important role in stabilizing Pt nanoparticles.

In this study, we used one-pot flash Joule heating (FJH) method to obtain high-quality graphene (Zhu et al., 2022), and synthesized graphene-supported platinum-nickel alloy nanoparticles (PtNi NPs) electrocatalysts (PtNi/graphene) *via* electrodeposition method as developed by Zhao et al. (2007a). The electrocatalytic characteristics of PtNi/graphene toward oxygen reduction reaction (ORR) were systematically investigated. Up to our knowledge, this technique is used for the first time for Pt-based alloy nanoparticle electrochemically deposited onto graphene materials. The properties of prepared catalyst are analyzed with transmission electron microscopy (TEM), Thermogravimetric analysis (TGA), X-ray diffraction (XRD), X-ray photoelectron spectra (XPS), and Raman spectroscopy. Finally, the electrochemical stability of PtNi/graphene upon accelerated degradation is also assessed.

## 2 Materials and methods

### 2.1 Materials

All of the chemicals are of analytical grade and used without further purification. Commercial references of Vulcan carbon-supported Pt electrocatalyst (50 wt%, TEC-10E50E) was purchased from TKK, Japan. Other reagents are as follows: isopropanol (IPA, >99.9%, analytical reagent grade, Kermel), Nafion D521 dispersion (5 wt%, EW = 1100, Ion Solution Inc), potassium tetrachloroplatinate (K_2_PtCl_4_, 98%, RHAWN), nickel dichloride (NiCl_2_, 99%, RHAWN), sulfuric acid (H_2_SO_4_, 95–98 wt%, analytical reagent grade, SCR, China), perchloric acid (HClO4, 70%, Sigma Aldrich). Argon and oxygen gases having high purity (>99.99%) were purchased from Yihong Gas Company, China. Deionized water (20 ± 1°C, pH 7, *ρ* = 18.3 MΩ/cm) was purified by passing through pure compact ultrapure water system (Arium mini, Sartorius).

### 2.2 Preparation of the Pt-Ni (2:1) alloy graphene-supported electrocatalyst

The electrodeposition approach is developed from previous study by Zhao et al. (2007a). Preparation of PtNi/graphene catalyst by electrochemical reduction—The process of electrochemical reduction and loading were carried out in a three-electrode cell controlled by a DH7003 workstation. A Pt wire (99.9%) was used as the counter electrode, and Ag/AgCl was used as the reference electrode. All potentials listed are referenced to the reversible hydrogen electrode (RHE). Graphene was functionalized and branched with many functional groups in 0.5M K_2_SO_4_ by using cyclic voltammetry 50 cycles with potentiodynamic scanning from -0.13–2.07 V *vs.* RHE at 200 mV s^−1^, Pt^2+^, and Ni^2+^ were combined with the functional groups of the graphene in the mixed solution of 2 mM K_2_PtCl_4_, 1 mM NiCl_2_, and 0.1M K_2_SO_4_ by cycling the potential between 0.57 and 1.57 V *vs.* RHE at 100 mV s^−1^ for 100 cycles. Finally, PtNi/graphene catalyst was formed on the surface of GC as Pt^2+^ and Ni^2+^ were reduced into nanoparticles and uniformly loaded on graphene in 0.1M H_2_SO_4_ by cycling from 0 to 1.27 V *vs.* RHE at 100 mV·s^−1^ for at least 30 cycles or more if the cyclic voltammetry curve had not yet reached a steady state.

### 2.3 Electrochemical measurement

The electrocatalysts were electrochemically characterized in a three-electrode cell with a rotating disk electrode (RDE) setup (Pine Instruments) controlled by a Donghua potentiostat DH7003). The Pt wire (99.9%, Alfa Aesar) was used as the counter electrode, and the Ag/AgCl (BASi) was used as the reference electrode. The Pt loaded (15 μg cm^−2^) glassy carbon (GC) disk (0.196 cm^2^, HTW) was immersed into 0.1 M HClO_4_ as the working electrode. The thin film catalyst layer on GC was formed by drop casting from a catalyst ink and drying under a flow of Ar. The catalyst ink was prepared by sonicating solid catalyst powder in a 4:1 H_2_O:IPA volume ratio solvent solution with concentration of 1 mg_catalyst_ ml^−1^. In order to well disperse and stabilized the catalyst particles on the GC surface, 0.5 µL of Nafion 5 wt% solution per mg of catalyst was added to the ink. Cyclic voltammograms (CVs) were performed with Ar purging at 294 K, with the potential scanned from 0.0 to 1.1 V *vs.* RHE at 20 mV s^−1^, and were used to determine the electrochemically active surface area of the Pt catalyst by integrating hydrogen desorption (∼0–∼0.35 V *vs.* RHE). ORR activities were measured in O_2_-saturated 0.1 M HClO_4_ at 294 K, with the potential scanned between 0.1 and 1.1 V vs. RHE at 20 mV s^−1^ at a rotation rate of 1600 rpm. All potentials are corrected for iR drop within the electrochemical cell.

### 2.4 Physicochemical characterization

High angle annular dark field scanning transmission electron microscopy (HAADF-STEM) images were taken of samples supported on lacey carbon grids in a FEI talos F200x G2 TEM/STEM operated at 200 keV. Energy dispersive spectroscopy (EDS) in the STEM mode was employed for elemental composition and distribution of the catalyst particles. Thermogravimetric analysis (TGA) of the catalysts was carried out under a mixed gas atmosphere (total flow: 25 ml min^−1^, O_2_:N_2_ = 1:4) at a constant rate of 10°C min^−1^, using a Netzsch STA 449 F3 system. The catalyst (≈10 mg) was loaded into an alumina crucible and heated from room temperature to 1000°C. X-ray diffraction (XRD) profiles were collected on Bruker D8 spectrometer with Cu Kα radiation (*λ* = 0.15406 nm). X-ray photoelectron spectra (XPS) were carried out using a Thermo Scientific K-Alpha X-ray photoelectron spectrometer. The binding energy was corrected using the C 1 s peaks (284.5 eV) as reference. Raman measurement was performed on a Renishaw in *via* Raman spectrometer with an excitation wavelength of 785 nm.

## 3 Results and discussion

**SCHEME 1:**
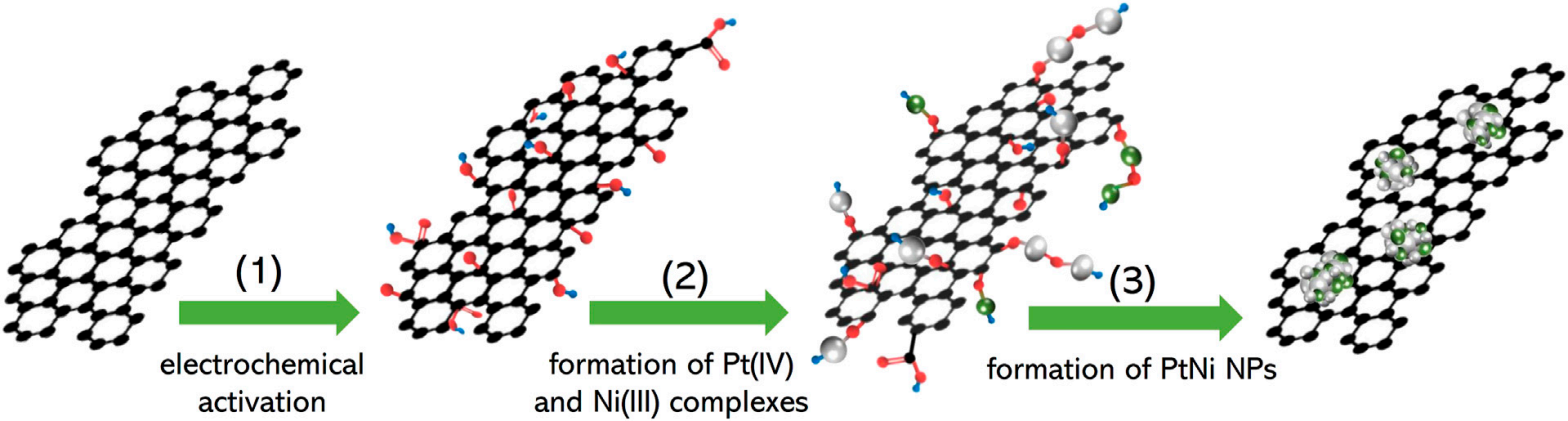
Schematic illustration of the electrodeposition process of PtNi NPs onto graphene according to previous work by Zhao et al. (2007b) Black, red, white, green and blue elemental ball represents atom carbon, oxygen, platinum, nickel and hydrogen, respectively.

**FIGURE 1:**
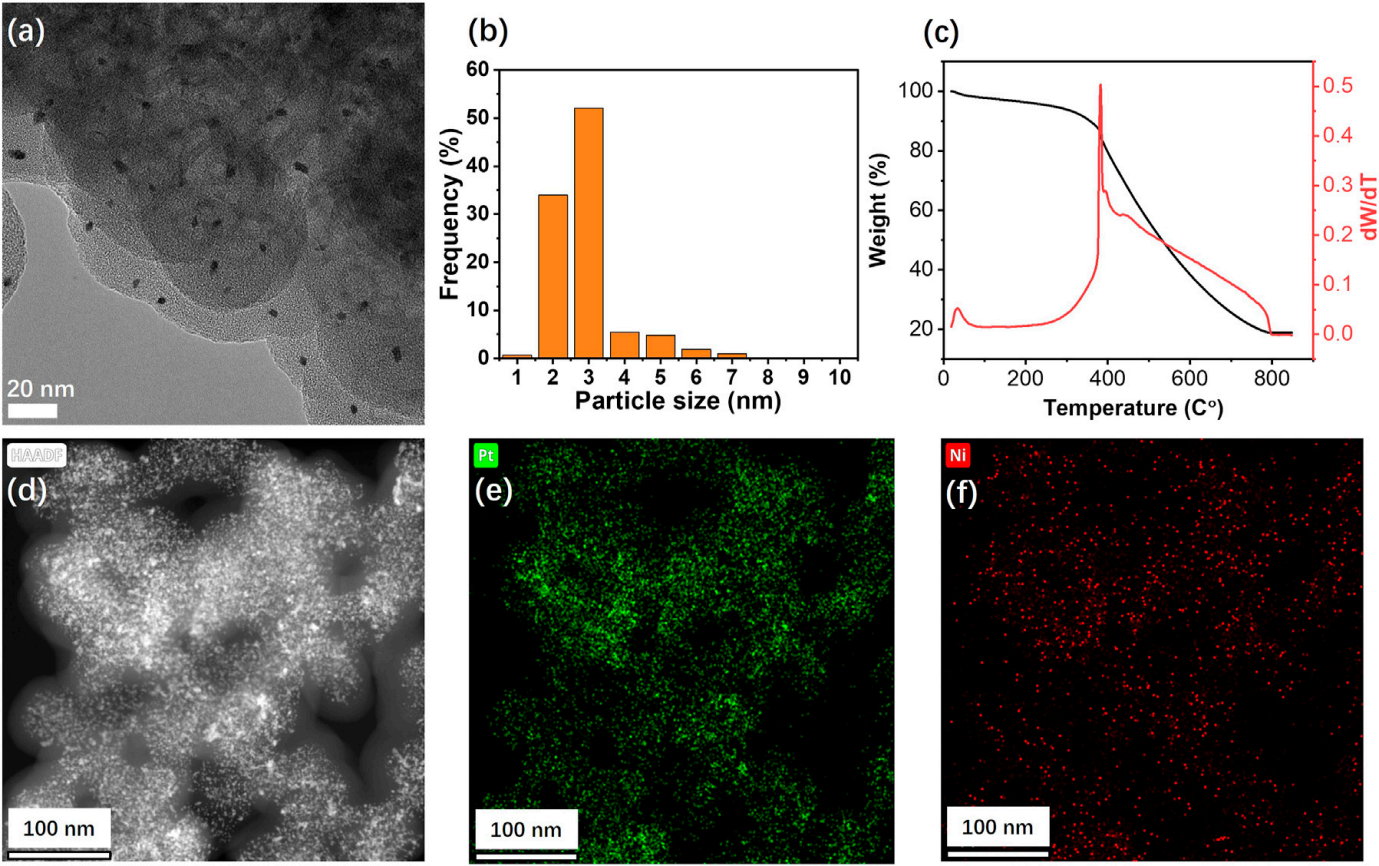
**(A)** HRTEM image, **(B)** particle size distribution diagram, and **(C)** thermogravimetric analysis of PtNi/graphene. **(D)** HAADF images and **(E)** Pt and **(F)** Ni EDS maps of Pt−Ni binary alloy nanoparticles supported on graphene.

The electrochemical deposition route of PtNi NPs on graphene is illustrated in Scheme 1, where the synthesis of the PtNi/graphene electrocatalyst was performed through an established three-step process (Zhao et al., 2007b): 1) electrochemical activation to generate oxide functional groups on graphene; 2) formation of complexes of Pt(IV) and Ni(III) on the graphene through oxidation of PtCl_4_^2−^ and Ni^2+^ from metallic salt solution; 3) conversion of the surface complexes to PtNi alloy nanoparticles through potential cycling. Figure 1A shows HRTEM image of graphene after electrochemical deposition of PtNi NPs with uniform size homogeneously decorated on the graphene. The mean size of the Pt NPs on graphene was estimated to be 2.9 nm (Figure 1B). STEM and corresponding elemental mapping demonstrate the homogeneous distribution of the PtNi NPs and the molar ratio of Pt to Ni was found to be near 2:1 by EDS, confirming that the platinum and nickel precursors have been electrochemically and fully reduced to PtNi NPs. (Figures 1D–F). This alloy NPs-decorated graphene shows great potential as electrocatalytic nanomaterials due to both accessible faces of the carbon materials (Geim, 2009). And the metal loading of 20 wt% on the graphene is determined by TGA (Figure 1C).

**FIGURE 2:**
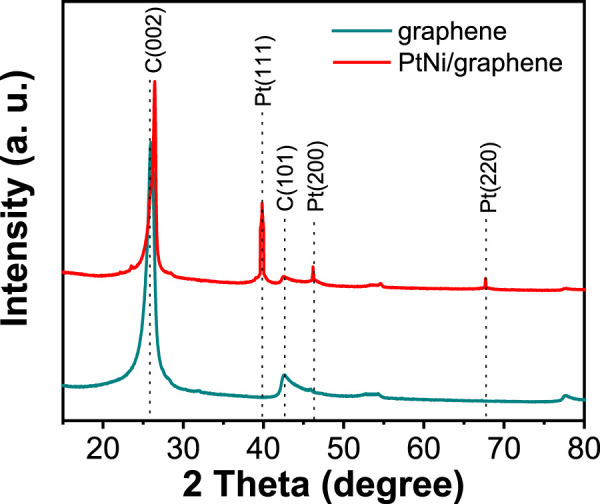
XRD patterns of bare flash graphene and as-made PtNi/graphene.

The XRD pattern of bare flash graphite obtained by FJH and as-made PtNi/graphene is shown in Figure 2. The 2θ values at 26.5° and 42.7° display a sharp C(022) peak along with a weak C(101) peak indicating the presence of turbostratic graphene support (Zhu et al., 2022). The turbostratic graphite structure is characterized by a two dimensional graphite structure in which the layers are misaligned to each other *via* translation or rotation while the interlayer spacing approaches that of crystalline graphite (0.335 nm) (Soin et al., 2011). After deposition of electrocatalyst nanoparticles, the diffraction peaks at 40.0° for Pt(111), 46.2° for Pt(200), and 67.5° for Pt(220) are observed, indicating the characteristic fcc platinum lattice (Niu et al., 2012). No characteristic peaks of Ni were detected suggesting that Pt is well alloyed with Ni (Suh et al., 2016). The slight positive peak shift of C(002) with PtNi incorporated can be attributed to the change of the interlayer distance in the graphene during electrochemical oxidation/reduction process (Niu et al., 2012).

**FIGURE 3:**
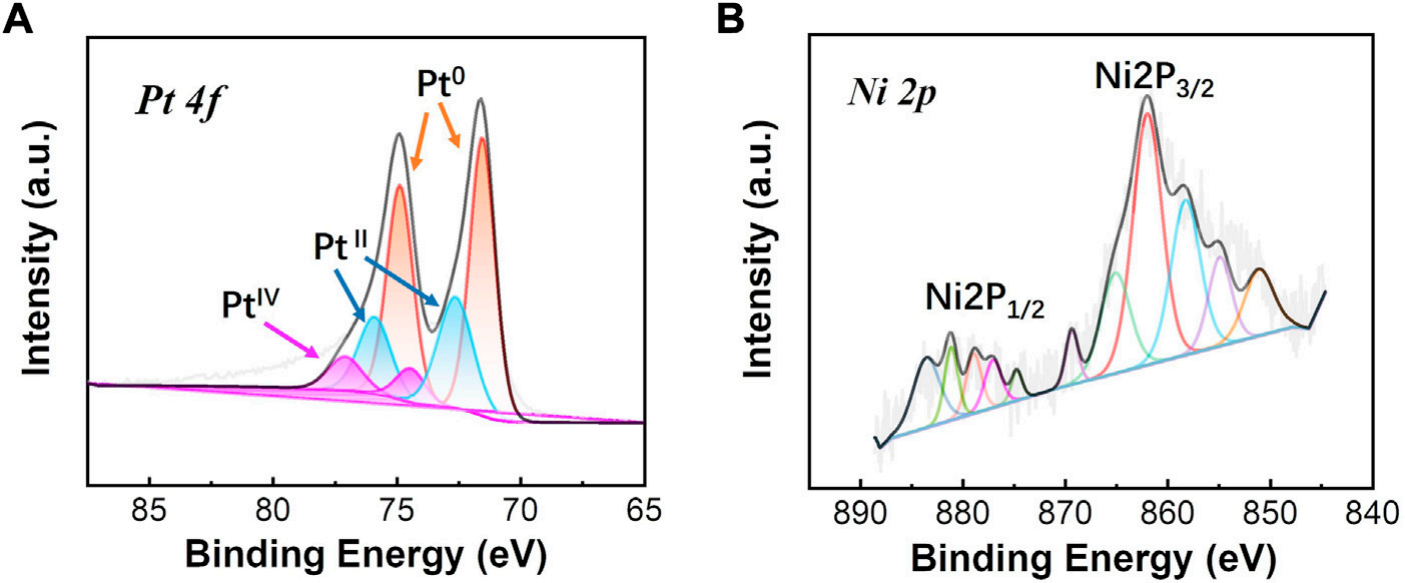
**(A)** Pt4f and **(B)** Ni2p XPS spectra profiles of as-made PtNi/graphene.

The formation and deposition of the PtNi NPs on graphene was further confirmed by XPS (Figure 3). Figure 3A shows Pt 4f XPS spectra which can be deconvoluted into Pt^0^, Pt^Ⅱ^, and Pt^Ⅳ^. The observed binding energy of Pt^0^ is 71.49 and 74.93 eV agreeing well with the reported value of Pt^0^ (Suh et al., 2016; Peng et al., 2017). The binding energy of Pt^Ⅱ^ and Pt^Ⅳ^ is observed to be 72.56 and 77.09 eV, respectively. The relative distribution of Pt^0^ specie is found to be ∼62 at% with the rest being present as oxides in oxidation states, suggesting the well metallic state of the Pt-based electrocatalysts. As shown in Figure 3B, metallic Ni and Ni oxide species were also observed indicating the main Ni 2P peaks which corresponds to Ni 2P_3/2_ and Ni 2P_1/2_, respectively (Yu et al., 2022). By mainly presenting in the Ni species based on the peak areas of Ni 2P, the presence of Ni oxide such as NiO and Ni(OH)_2_ can promote an increase of metallic Pt and a decrease of Pt oxides states due to the alloying effect of Ni on Pt (Wang et al., 2010). The presents of Ni oxide may result from the electrochemical oxidation during electrodeposition process of PtNi NPs.

**FIGURE 4:**
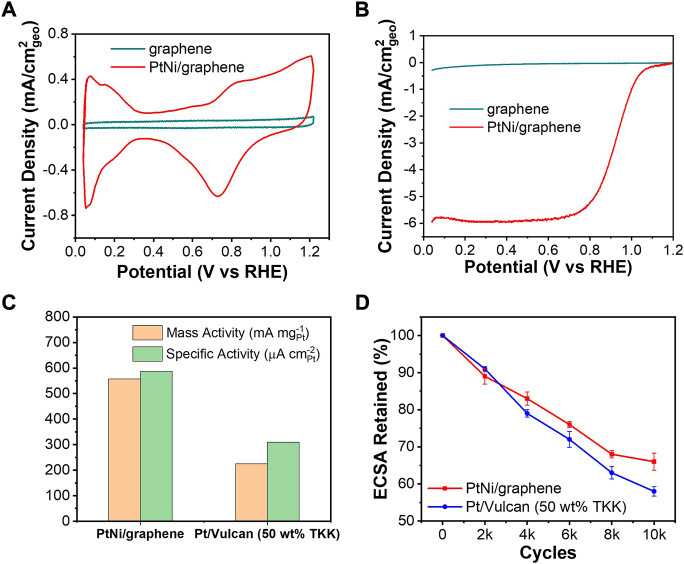
**(A)** Cyclic voltammograms and **(B)** ORR polarization curves of bare flash graphene and as-made PtNi/graphene. **(C)** Kinetic activities at 0.9 V *vs.* RHE and **(D)** ECSA retained during AST of PtNi/graphene and commercial reference Pt/Vulcan carbon.

Figure 4A shows the cyclic voltammograms of the bare graphene and resulting PtNi/graphene in 0.1 M HClO_4_. Representing hydrogen adsorption/desorption process, the reversible hydrogen underpotential deposition (H_UPD_) in an electrochemical system can be used to determine electrochemical active surface area (ECSA), which is essential for understanding the utility of Pt by evaluating the number of available electrochemically active sites (VlietVan Der et al., 2012). Comparing with bare flash graphene, it can be observed that the H_UPD_ peaks between 0.05 and 0.4 V *vs.* RHE and oxidation/reduction between 0.6 and 1.2 V *vs.* RHE of the Pt surface are clearly presented for PtNi/graphene, indicating the presence of active Pt (Kocha et al., 2017). ECSA can be calculated from H_UPD_ charges and the amount of Pt loading on the electrode (Eq. 1): (VlietVan Der et al., 2012)

$$ECSA=Q_{H}/\left(Pt\;loading\times 0.21\right)$$ (1)

where Q_H_ is the average charge of hydrogen adsorption/desorption (mC), and the value of 0.21 is known as the charge for the monolayer if hydrogen adsorption on the Pt surface. The corresponding ECSA of Pt is determined to be 73.9 m^2^/g for the PtNi/graphene which is comparable to commercial reference of Pt-based electrocatalyst supported by Vulcan carbon (Garsany et al., 2014). The obtained well-defined hydrogen adsorption/desorption characteristics can be contributed by the fact of the small size of PtNi NPs dispersed uniformly on graphene planes (Figure 1A).

The ORR activities of PtNi/graphene were characterized by a RDE setup in 0.1 M HClO_4_ as shown in Figure 4B. The current density for reduction of oxygen was significantly increased with deposition of PtNi NPs on the graphene, exhibiting characteristic Pt electrocatalytic ORR behaviors. Koutechy-Levich (K-L) Equation (Eq. 2) was applied to quantitatively evaluate the ORR activities (Miah and Ohsaka, 2009)

$$\frac{1}{j}=\frac{1}{j_{d}}+\frac{1}{j_{k}}$$ (2)

where *j* is the measured current density (mA/cm^2^), *j*_d_ is the diffusion limiting current density under the potential region of 0.2–0.65 V *vs.* RHE, and *j*_k_ is the kinetic current density which can be obtained based on K-L equation to adjust for mass transport limitations. (Miah and Ohsaka, 2009) As shown in Figure 4C, both mass activities and specific activities of PtNi/graphene and commercial reference of Vulcan carbon-supported Pt electrocatalyst were evaluated based on the calculated *j*_*k*_ at 0.9 V *vs.* RHE. The PtNi NPs supported on graphene exhibited a substantially higher both mass activity and specific activity compared to Pt electrocatalyst supported on Vulcan carbon. In addition to alloy effect and electronic ligand effect from the second transition metal Ni,(Stamenkovic et al., 2007) the outstanding ORR performance can be explained by the decreased charge transfer resistance (*R*_CT_) due to excellent electrical conductivity of the graphene. Wang et al. (2010) EIS technique was conducted to study the *R*_CT_ of graphene obtained using FJH method in our previous work, (Zhu et al., 2022) and a near-vertical curve in the low-frequency region and a semicircle in the high-frequency region for the graphene was observed, indicating the low *R*_CT_.

**FIGURE 5:**
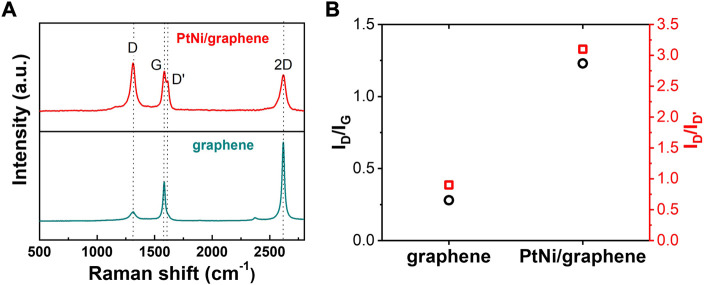
**(A)** Raman spectra of bare flash graphene and as-made PtNi/graphene. **(B)** Comparison of *I*_D_/*I*_G_ and *I*_D_/*I*_D’_ values of bare flash graphene and as-made PtNi/graphene.

Moreover, It is reported that the density of monovacancy site on graphene plays key role in its electrocatalytic performance (Lim and Wilcox, 2012). The representative Raman spectrums of flash graphene nanomaterial and PtNi/graphene obtained through electrodeposition method are shown in Figure 5A. A sharp G band peak at ∼1585 cm^−1^ and 2D band peak at ∼2620 cm^−1^ were clear observed for graphene, indicating its high degree of graphitization (Zhu et al., 2022). For PtNi/graphene, rather than graphene from which D band was barely found, we can see a sharp and high D band and defect-induced D′ peak at ∼1320 cm^−1^ and ∼1620 cm^−1^, respectively. The intensity ratio of D and D′ band (*I*_D_/*I*_D’_) is commonly used to illustrate the defect nature in the atomic structure of the graphene (Eckmann et al., 2012). As displayed in Figure 5B, the value of *I*_D_/*I*_D’_ for PtNi/graphene is 3.1, which is much higher than that of graphene (0.9), suggesting the formation of many structural defects or disorders on the graphene support where PtNi NPs are deposited(Zhu et al., 2022). The conclusion is also supported by analyzing the intensity ratio of D and G band (*I*_D_/*I*_G_) (Figure 5B). This defects evolution in graphene can be ascribed to the process of PtNi NPs electrodeposition during which electrochemical cycling induces and enables additional defects to facilitate metallic ions diffusion through the graphene layer (Jaber-Ansari et al., 2014). Using density functional theory (DFT) modelling and Raman spectra, (Jaber-Ansari et al., 2014) has revealed that, upon potential cycling, defectivity is initiated with vacancy formation and chemical functionalization through the interaction between graphene and absorbates such as metallic ions and oxygen. DFT also indicates that graphene defect sites lower the activation energy of oxygen dissociation and reduce the stability of intermediate HO* species, thermodynamically driving ORR toward 4e^−^ pathway and facilitating its kinetic activities (Figure 4C). (Lim and Wilcox, 2012)

An accelerated stability test (AST) of PtNi/graphene was also performed by cycling potentials between 0.6 and 1.1 V *vs.* RHE in 0.1 M HClO_4_ at 50 mV s^−1^ under Ar atmosphere as suggested in our previous work (Li et al., 2017), and the ECSA was evaluated for every 2000 cycles. As shown in Figure 4D, PtNi/graphene performs with better ECSA retention than commercial references of Pt/Vulcan. This general improvement is expected as the defect sites of graphene support (Figure 5A) reserves strong interaction with PtNi NPs(Lim and Wilcox, 2011), preventing sintering of alloy nanoparticles and extending its ECSA retention. Further study needs to be undertaken to investigate the effects of graphene defectivity to electrocatalytic activity and stability.

## 4 Conclusion

In summary, novel Pt-Ni binary alloy nanoparticles electrocatalysts supported on graphene nanomaterials were successfully prepared by electrodeposition method. The PtNi NPs with sizes of ∼3 nm uniformly dispersed on graphene surface and loading of metals was determined to be 20 wt%. The resultant PtNi/graphene exhibits excellent electrocatalytic activity and stability toward the reduction of oxygen. In addition to the improved surface electronic properties due to characteristic of graphene, the formation of structural defects and disorders on graphene support during electrodeposition process can also attribute to the electrocatalytic performance of PtNi NPs. These results indicate that graphene nanomaterials could be a good candidate as a supporting material of electrocatalysts, and electrodeposition method is promising for the preparation of high-performance graphene-supported alloy electrocatalysts.

## Data Availability

The original contributions presented in the study are included in the article/supplementary material, further inquiries can be directed to the corresponding authors.
